# Development and application of Adverse drug reactions reports QUality Algorithm (AQUA-12) score: a single-centre quality improvement initiative

**DOI:** 10.1007/s00228-023-03457-9

**Published:** 2023-02-20

**Authors:** Ar Kar Aung, Celia M. Zubrinich, Michelle S. Y. Goh, Benjamin Snyder, Mei Jie Tang, Cindy Y. L. Khu, Jennifer I. Lee, Linda V. Graudins

**Affiliations:** 1 Department of General Medicine, Alfred Health, 55 Commercial Road, Melbourne, VIC 3004 Australia; 2 School of Public Health and Preventive Medicine, Monash University, Melbourne, Australia; 3 Allergy, Immunology and Respiratory Medicine, Alfred Health, Melbourne, Australia; 4 Department of Dermatology, Alfred Health, Melbourne, Australia; 5 Therapeutic Goods Administration, Canberra, Australia; 6 Department of Infectious Diseases, Alfred Health, Melbourne, Australia; 7 Department of Medicine, New York-Presbyterian Hospital, New York, USA; 8 Quality Improvement Academy, Weill Cornell Medicine, New York, USA; 9 Department of Pharmacy, Alfred Health, Melbourne, Australia

**Keywords:** Adverse drug reactions, Quality improvement, Quality algorithm, Quality assessment, Medication safety

## Abstract

**Purpose:**

To develop a reliable assessment tool to monitor the quality of adverse drug reaction (ADR) reports and evaluate its performance within a quaternary hospital setting.

**Methods:**

Adverse drug reactions report QUality Algorithm (AQUA-12) was developed by a multidisciplinary team with the expertise in the management of ADRs. The design was based on data elements required to establish medication causality. Inter-rater reliability of AQUA-12 was evaluated over three rounds in two phases: development and prospective evaluation phases, by independent assessors both internal and external to the institutional ADR review processes. The characteristics and quality of ADR reports were subsequently assessed, and potential factors contributing to low-quality reports were identified.

**Results:**

A total of 70 ADR reports were assessed, 20 in development and 50 in evaluation phases. The inter-rater reliability of AQUA-12 was found to be excellent in all three rounds (Cronbach’s alpha of  ≥ 0.9, *p* < 0.001 for all). Approximately one in five reports concerned immediate hypersensitivity reactions while delayed hypersensitivity reactions constituted 60% of all reactions. AQUA-12 identified 18 (25.7%) reports as ‘low-quality’ with a score of  < 10. Identification of suspected medications (37.1%), description of index ADR (27.1%), and key events (ADR narrative, 35.7%) were the top data elements incomplete or missing from all reports. Univariable analyses identified the severity of the reaction as a factor associated with low quality of reports (*p* = 0.008).

**Conclusions:**

AQUA-12 is a practical and highly reliable assessment tool that can be utilised in hospital settings to regularly monitor the completeness of ADR reports to guide quality improvement initiatives.

## Introduction

Adverse drug reactions (ADR) pose a significant burden to healthcare, accounting for 3 to 5% of hospital admissions [1, 2]. It is estimated that 10–17% of hospitalised patients experience an ADR, which may result in up to a two-fold increase in the length of hospital stay [1, 3, 4]. Severe ADRs may have a long-lasting physical and psychological impact on patients [5, 6]. Medication-related hospital admissions cost AU $1.2 billion annually, yet a substantial proportion of ADRs are preventable with appropriate management [7].

An important element of ADR management within a hospital setting is the documentation and reporting of ADR episodes, facilitated by a centralised internal review process, with subsequent reporting to pharmacovigilance authorities. Main aspects of ADR reporting that are often discussed are the under-reporting of ADRs and ways to increase the quantity of reports. However, the quality of ADR reports is equally important as a minimum dataset is required to assess medication reaction causality. This process requires the application of technical skills and knowledge by reporting healthcare professionals, including knowledge of ADR syndromes and pathogenesis, recognition of underlying comorbidities and concurrent conditions mimicking an ADR, familiarity with pharmacologic profiles of medications and drug interactions, and ability to construct a relevant medication timeline [8]. An incomplete or a low-quality report undermines the strength of the association between a medication and a reaction. Comprehensive and accurate risk communication, with recommendations to patients and healthcare professionals is not possible without a high-quality assessment [9].

It is recognised that ADR reporting processes are subject to large practice variations, often influenced by healthcare providers’ attitudes and beliefs, knowledge and skills, clinical expertise and practice settings, as well as familiarity with local protocols and reporting requirements by authorities [10]. Such unintended variations potentially compromise patient safety and diminish the quality of care. Appropriate clinical indicators to measure and monitor the quality in ADR management processes within hospital settings remain ill-defined. Moreover, currently available tools that assess the quality of ADR reports are almost exclusively designed for use in the research setting [10]. A rapid and reliable assessment tool for pragmatic application in daily clinical settings within hospital environments would facilitate quality monitoring and process improvement.

In this quality improvement initiative, we developed a pragmatic scoring system, Adverse drug reactions reports QUality Algorithm (AQUA-12), that enables regular monitoring of the quality of ADR reporting processes in hospital settings. The primary aim was to evaluate the performance of AQUA-12 during the development and application phases. Secondary aims were to evaluate the quality of ADR reports and to determine potential factors contributing to low-quality reports to target improvements.

## Methodology

### Setting

AQUA-12 was designed by members of the Adverse Drug Reaction Review Committee (ADRRC) at Alfred Health in Melbourne, Australia. Alfred Health is a quaternary, university-affiliated health institution that provides, among many clinical services, specialist care in transplantation, human immunodeficiency virus (HIV) infection, cystic fibrosis, haemophilia, trauma and burns. The ADRRC is a multidisciplinary group consisting of senior pharmacists in medication safety and medicines information, and specialist physicians in allergy/immunology, dermatology, clinical pharmacology, infectious diseases and internal medicine. Around 200 ADR episodes per year are reported to ADRRC. The committee meets every 2 weeks to discuss ADR reports, assign causality, organise further referrals as required (e.g. allergy services) and provide recommendations regarding future medication management. All healthcare professionals within the institution are encouraged to submit ADR reports; approximately 85% of the reports are submitted by hospital pharmacists [11]. The reporting system is predominantly via an electronic form embedded in the electronic medical record (EMR).

The need to design a scoring system to monitor the quality of ADR reports was first identified in mid-2021 when ADRRC began to develop an ADR education program for hospital pharmacists and junior doctors. This sought to improve the knowledge, technical skills and competency required to conduct a comprehensive assessment of an ADR episode. The tool was intended to assess the completeness of information in submitted ADR reports and would be a surrogate marker to assess practical knowledge and technical attributes. Hence, we intend to measure improvement over time, following the planned educational program.

### Development phase

The primary objectives of AQUA-12 were to assess the completeness of data to allow the ADRRC to assess causality and provide effective risk communication to patients. The emphasis was placed on the following data elements for scoring: (i) previous ADR history, (ii) diagnosis or description of actual ADR, (iii) description of key events concerning ADR (i.e. the narrative), (iv) list of suspected medications, (v) consideration of medication timeline relevant to the nature of ADR, (vi) management of ADR episode and (vii) outcome/sequelae. The rationale behind the inclusion of each data element is summarised in Table 1. The data elements also reflect data fields in the ADR reporting form that are required to be completed by healthcare professionals and excluding those automatically populated by the EMR, such as patient details, reporter details. Each data element is assigned a maximum score of 2, except for the relevant medication timeline (temporality) and management, which are assigned a score of 1 each, giving a total score of 12.

**Table 1:** Included data elements in AQUA-12 quality assessment tool and underlying principles

Data elements	Specific information required	Rationale for inclusion
Previous ADR history	Implicated generic medication names (and active ingredients if known) Description/diagnosis of reaction	Previous reaction from exposure to same or structurally similar medications increases strength of causal association
		Detailed description/diagnosis of reaction is important to determine the exact nature of past ADR (i.e. intolerance vs. allergic, benign vs. of clinical concern, etc.)
Actual reaction	Detailed description or diagnosis of index reaction	Detailed description or diagnosis provides information regarding the exact nature of current reaction
		This influences management advice regarding avoidance or rechallenge of suspected medications (e.g. complete avoidance in SCARs and anaphylaxis vs. possible rechallenge or treat-through in mild MPE)
		Ambiguous descriptions without details (e.g. “rash”) are unhelpful in clinical decision making
Description of key events	Narrative surrounding the reaction which include: onset, offset, evolution, co-morbidities that may mimic or contribute to the reaction, medication timelines, presence of drug-drug interactions, relevant investigations or information (previous tolerant exposure, re-challenge etc.)	Detailed information regarding ADR onset, evolution, offset, and timelines aids in causality assessment
		Information on relevant investigations (e.g. skin biopsy, presence of eosinophilia, organ dysfunction) aids in confirming the diagnosis as well as causality assessment
		Information on co-morbidities is helpful in considering non-ADR related differential diagnoses or underlying factors (i.e. renal impairment) that might have contributed to ADR
		Highlighting relevant drug-drug interactions is helpful in determining preventable factors
Suspected medications	A list of medications that may have caused ADR. A complete set of information include: generic medication names (and active ingredients if known), indications, date commenced, date ceased, route, dosage and frequency	Comprehensive review of medication list, including over the counter medications, in a patient with ADR aids in identification of a set of possible culprit medications
		Indications, commencement and cessation dates, route, dose, frequency helps determine most likely medication(s) causing the reaction
Timeline relevant to the nature of ADR	Based on the nature of ADR (i.e. Type A or Type B, immediate or delayed hypersensitivity, etc.) all medication(s) that fall within relevant time frame to be included	Recognition of timelines specific to the nature of ADR is important to avoid inadvertent omission of medications that may be implicated in the reaction or over-inclusion of medications as suspects when, in actual fact, they fall outside the time frame
		This avoids mislabelling of implicated medications
Management of reaction	Information on how the ADR episode is managed, including: dose reduction, cessation, antidote treatment, re-challenge, monitoring	Management narrative aids in causality assessment and helps strengthen causality
Outcome/Sequalae	Information regarding severity and outcome from the ADR episode	This influences management advice regarding avoidance, referral for further investigation/ evaluation or judicious rechallenge of suspected medications
		Also determines if certain reports need to be forwarded to the pharmacovigilance authority (i.e. Therapeutic Goods Administration)

*MPE* Maculopapular exanthems, *SCARs* severe cutaneous adverse drug reactions

In Round 1 of this cross-sectional study, the first version of AQUA-12 was evaluated using 20 randomly selected ADR reports submitted to ADRRC between 11th January and 4th June 2021. Two ADRRC members (AKA, LG) and one junior doctor, who was not part of the ADRRC, independently assessed the reports retrospectively for completeness after the ADRRC review had been conducted. The scores were then analysed for inter-rater correlation. Any reports displaying discrepancy in total scores between assessors by more than two points were identified and reasons behind differences were discussed. The scoring criteria and wording were refined to make them more concise and easily interpretable.

In Round 2, a revised version of AQUA-12 was retrospectively and independently evaluated using the same 20 ADR reports as above, but by a different set of ADRRC members consisting of one clinical pharmacologist (BS), one dermatologist (MG) and one allergist/immunologist (CZ). External to the ADRRC, a clinical pharmacologist and a junior doctor who were not familiar with the current ADRRC review processes, also independently evaluated the reports. Inter-rater correlation analysis was conducted using the scores of 5 assessors from Round 2. The tool was then further refined to improve functionality.

In both rounds during the development phase, final ADR diagnoses and management recommendations by ADRRC, as well as further clinical information, were made available to the independent assessors scoring the reports.

The final version of AQUA-12 derived from the above process is provided in Table 6.

### Evaluation phase

In this phase, AQUA-12 was used to assess 50 consecutive reports submitted to ADRRC between 1st Jan 2022 to 18th April 2022. Reports were scored independently by AKA and LG, and inter-rater correlation analysis was conducted. The first assessor (AKA) prospectively scored the ADR reports in a blinded manner prior to the scheduled ADRRC review fortnightly. The second assessor (LG) independently scored after further information (diagnosis, investigations and recommendations) was made available post-ADRRC review of the reports.

### Data variables and outcomes

The following data variables concerning all ADR reports were extracted from electronic medical records: vocation of reporters, treating clinical unit, reaction type, reaction severity and implicated medication classes. Reaction types and implicated medication classes were classified according to the methodology previously described in a publication by ADRRC to maintain consistency [11]. Outcomes of interest were as follows: (i) inter-rater correlation of AQUA-12 scores in both rounds of the development phase and in the prospective evaluation phase, (ii) proportion of high-quality reports using AQUA-12 tool and (iii) factors that may be associated with low-quality reports.

### Data analysis

Summary statistics for discrete variables are presented as counts and proportions. Inter-rater correlation analysis results are presented as intraclass correlation coefficient (Cronbach’s alpha) with 95% confidence intervals. Univariable analyses were conducted to identify any factors that may be associated with the poor quality of reports. For differences in proportions between groups, Fisher’s exact or chi-square tests were conducted, and statistically significant results are presented as a two-tailed *p* value of < 0.05. Data analysis was done using SPSS version 28 (IBM Corporation, Armonk, NY, USA).

### Ethics approval

Approval to conduct this study as a low-risk research project was granted by the Alfred Health Human Research and Ethics Committee (project number: 726/21).

## Results

A total of 70 ADR reports were included in the final analysis: 20 from the development phase and 50 from the prospective evaluation phase. The characteristics of ADR reports are displayed in Table 2. Most reports were submitted by clinical pharmacists and originated within medical units. Immediate hypersensitivity reactions accounted for one in five reports, and delayed hypersensitivity reactions constituted 60% of all reactions. The majority of reactions reported were moderate to severe in nature, involving predominantly antimicrobials. There were no statistically significant differences in ADR report characteristics between the two phases.

**Table 2:** ADR report characteristics

Characteristics	Total (*N* = 70)	Development phase (*N* = 20)	Prospective evaluation phase (*N* = 50)	*p*-value
Reporter, *n* (%)				
Pharmacist	60 (85.7)	18 (90)	42 (84)	0.71^a^
Doctor	10 (14.3)	2 (10)	8 (16)	
Reporting unit, *n* (%)				
Medical	37 (52.9)	12 (60)	25 (50)	0.19^b^
Surgical	14 (20)	5 (25)	9 (18)	
ED	10 (14.3)	0 (0)	10 (20)	
Other	9 (12.9)	3 (15)	6 (12)	
Reaction type, *n* (%)				
Immediate hypersensitivity	13 (18.6)	4 (20)	9 (18)	0.09^b^
Delayed hypersensitivity, non-SCAR	18 (25.7)	2 (10)	16 (32)	
Delayed hypersensitivity, single-organ involvement	14 (20)	2 (10)	12 (24)	
Delayed hypersensitivity, SCAR	10 (14.3)	5 (25)	5 (10)	
Non-immunological	7 (10)	4 (20)	3 (6)	
Other	8 (11.4)	3 (15)	5 (10)	
Severity, *n* (%)				
Mild	7 (10)	1 (5)	6 (12)	0.48^b^
Moderate	33 (47.1)	12 (60)	21 (42)	
Severe	16 (22.9)	2 (10)	14 (28)	
Life-threatening	7 (10)	3 (15)	4 (8)	
Fatal	2 (2.9)	0 (0)	2 (4)	
Not recorded	5 (7.1)	2 (10)	3 (6)	
Medication classes, *n* (%)	(*N* = 112)	(*N* = 31)	(*N* = 81)	
Anaesthetic agents	3 (2.7)	1 (3.2)	2 (2.5)	0.09^b^
Antiemetics	1 (0.9)	0 (0)	1 (1.2)	
Antiepileptics	3 (2.7)	0 (0)	3 (3.7)	
Antihypertensives	2 (1.8)	0 (0)	2 (2.5)	
Antimetabolites	3 (2.7)	2 (6.6)	3 (3.7)	
Antimicrobials	70 (62.5)	24 (77.4)	46 (56.8)	
Iron formulations	2 (1.8)	0 (0)	2 (2.5)	
NSAIDs	3 (2.7)	0 (0)	3 (3.7)	
Opioids	4 (3.6)	3 (9.7)	1 (1.2)	
Radiocontrast agents	4 (3.6)	1 (3.2)	3 (3.7)	
Others	15 (13.4)	0 (0)	15 (18.5)	

*ED* emergency department, *NSAIDs* non-steroidal anti-inflammatory agents, *SCAR* severe cutaneous adverse drug reaction

^a^Fisher’s exact test

^b^chi-square test

Table 3 shows the results of interrater correlation analysis from both the development and prospective evaluation phases. AQUA-12 yielded a high degree of correlation among assessors with Cronbach’s alpha value of ≥ 0.90 in all rounds of evaluation. Detailed results on mean scores by individual assessors and inter-item correlation matrices are provided in Tables 7, 8, and 9.

**Table 3:** Inter-rater correlation analysis for development and prospective evaluation phases of AQUA-12

Phase	Number of reports assessed	Number of assessors	Intraclass correlation coefficient (Cronbach’s alpha)	95% confidence interval	*p*-value
Development – round 1	20	3	0.90	0.80–0.96	< 0.001
Development – round 2	20	5	0.94	0.88–0.97	< 0.001
Prospective evaluation	50	2	0.90	0.83–0.95	< 0.001

Overall, 52 (74.3%) of reports were of high quality (defined as score of ≥ 10) with 30 (42.9%) reports scoring a possible maximum score of 12 (Table 4). ‘Suspected medication’ (37.1%), ‘Description of key events’ (35.7%) and ‘Actual reaction’ (27.1%) were data elements that were less frequently completed or partially completed.

**Table 4:** Quality of ADR reports and breakdown of incomplete components

Characteristics	Development phase (*N* = 20)	Prospective evaluation phase (*N* = 50)	Total (*N* = 70)
Quality of reports, *n* (%)			
Excellent (score 12)	9 (45)	21 (42)	30 (42.9)
Good (score 10–11)	4 (20)	18 (36)	22 (31.4)
Moderate (score 8–9)	4 (20)	8 (16)	12 (17.1)
Poor (score ≤ 7)	3 (15)	3 (6)	6 (8.6)
Components not or only partially completed, *n* (%)			
Previous ADR history	5 (25)	7 (14)	12 (17.1)
Actual reaction	5 (25)	14 (28)	19 (27.1)
Description of key events	7 (35)	18 (36)	25 (35.7)
Suspected medications	8 (40)	18 (36)	26 (37.1)
Timeline relevant to ADR	3 (15)	3 (6)	6 (8.6)
Management of reaction	2 (10)	4 (8)	6 (8.6)
Outcome/sequelae	3 (15)	3 (6)	6 (8.6)

*ADR* adverse drug reaction

Table 5 compares the characteristics between high- and low-quality ADR reports as determined by the AQUA-12 tool. Statistically significant differences in proportions of mild or undocumented severity of reactions were noted between high- and low-quality reports (5.8% high quality vs. 22.2% low quality for mild severity reactions, and 1.9% high quality vs. 22.2% low quality for reactions of undocumented severity). No notable differences exist between reporter and reaction types between the high- and low-quality ADR reports.

**Table 5:** Differences in characteristics between high quality (AQUA-12 score ≥ 10) and lower quality (score < 10) reports

Characteristics	High quality (*N* = 52)	Low quality (*N* = 18)	*p*-value
Reporter, *n* (%)			
Pharmacist	45 (86.5)	15 (83.3)	0.71^a^
Doctor	7 (13.5)	3 (16.7)	
Reaction type, *n* (%)			
Immediate hypersensitivity	7 (13.5)	6 (33.3)	0.13^b^
Delayed hypersensitivity, non-SCAR	14 (26.9)	4 (22.2)	
Delayed hypersensitivity, single-organ involvement	14 (26.9)	0 (0)	
Delayed hypersensitivity, SCAR	7 (13.5)	3 (16.7)	
Non-immunological	5 (9.6)	2 (11.1)	
Other	5 (9.6)	3 (16.7)	
Severity, *n* (%)			
Mild	3 (5.8)	4 (22.2)	**0.008**^b^
Moderate	29 (55.8)	4 (22.2)	
Severe	13 (25)	3 (16.7)	
Life-threatening	5 (9.6)	2 (11.1)	
Fatal	1 (1.9)	1 (5.6)	
Not recorded	1 (1.9)	4 (22.2)	

*SCAR* severe cutaneous adverse drug reaction

^a^Fisher’s exact test

^b^chi-square test

**Table 6:** Final version of Adverse drug reactions reports QUality Algorithm (AQUA-12) quality assessment tool. ADR Reports Quality Algorithm (AQUA12) version 2, 16.08.21

**Data elements**	**Definitions**	**Score**	**Maximum score (total = 12)**
**Previous ADR history**			
• Not elicited	No attempt made to record^a^	0	2
• Partially elicited	Mentions medication(s) only. No or ambiguous description of reaction provided (e.g. *rash*)	1	
• Fully elicited	Mentions medication(s) and reaction description/diagnosis included. If not known, states 'nil known or NKDA'	2	
**Actual reaction**			
• Not completed	No attempt made to record^a^	0	2
• Partially completed	Provides ambiguous or simple description only (e.g. *rash*)	1	
• Completed	Where applicable, states diagnosis clearly (e.g. *anaphylaxis*). If diagnosis not available, states key features and/or provide relevant negatives (e.g. *hypotension without associated systemic features of anaphylaxis; maculopapular drug eruption without systemic symptoms and internal organ involvement*)	2	
**Description of key events**			
• Not completed	No attempt made to record^a^	0	2
• Partially completed	Provides some account of reaction. Fails to note relevant comorbidities or predisposing factors pertaining ADR pathogenesis, diagnosis and/or causality (e.g. *records drug eruption but fails to note fevers, liver and renal function derangements for suspected DRESS*)	1	
• Completed	Provides detailed account of reaction. Also notes co-morbidities or predisposing factors that may have contributed to ADR and/or drug interactions and/or timeline supporting causality and/or cross-reactivity and/or past exposure (e.g. *patient has suspected flare of graft vs. host disease as a differential; patient tolerated other beta-lactams such as ceftriaxone during recent admission; patient has concurrent renal impairment that may have affected medication levels*)	2	
**Suspected medications**			
• Not completed	No attempt made to record^a^	0	2
• Partially completed	Highlights too few or too many medications, including unlikely medications commenced after reaction onset. Any information regarding indications, date commenced, date ceased, route, dosage, frequency is missing	1	
• Completed	Highlights all likely culprit medications, indications, date commenced, date ceased, route, dosage, frequency	2	
**Timeline relevant to the nature of ADR**^b^			
• Not considered	Medications that were commenced within time frame relevant to the nature of reaction NOT considered, or medication commenced outside relevant time frame included	0	1
• Considered	Accounts for relevant time frame between medication commencement, cessation and onset of reaction (e.g. *up to 8 weeks for delayed hypersensitivity reactions; within 2 h for anaphylaxis*)	1	
**Management of reaction**			
• Not completed	No attempt made to record^a^	0	1
• Completed	Includes management information regarding any of dose reduction, cessation, antidote treatment, re-challenge, monitoring	1	
**Outcome/sequelae**			
• Not completed	No attempt made to record^a^	0	2
• Partially completed	Records either outcome from the reaction or severity^c^ only	1	
• Completed	Records both outcome and severity^c^ (e.g. *response to therapy; not yet recovered; alternative treatment plan*)	2	

^a^If information is missing or states ‘please see medical records’, score ‘0’ as ADR reports are meant to serve as standalone documents

^b^The timeline is based on the nature of ADR (e.g. Type A vs. Type B, immediate vs. delayed) and the pharmacokinetic/pharmacodynamic profile of medications under consideration. Underlying basic knowledge in these areas is assumed

^c^Severity is recorded as mild, moderate, severe, life-threatening or fatal. Presentation to the Emergency Department is not a marker of severity

**Table 7:**
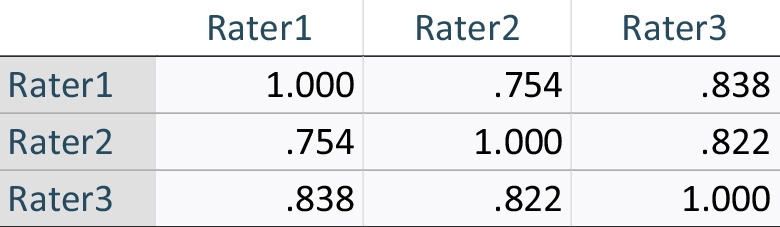
Inter-item correlation matrix for development phase, round 1, of evaluation of AQUA-12 tool

**Table 8:**
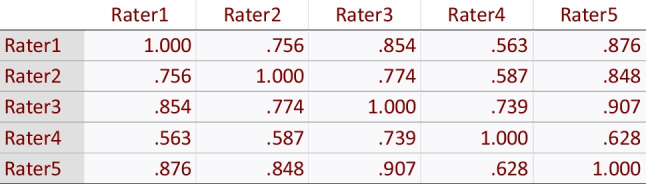
Inter-item correlation matrix for development phase, round 2, of evaluation of AQUA-12 tool

**Table 9:**
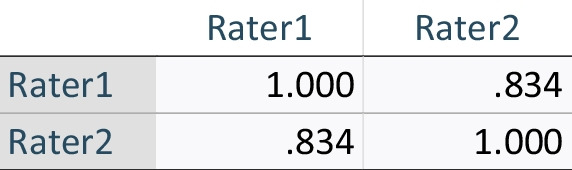
Inter-item correlation matrix for prospective evaluation phase, round 3, of evaluation of AQUA-12 tool

## Discussion

In the present study, we developed a tool that can be routinely utilised in hospital settings to monitor the quality and completeness of ADR reports. AQUA-12 was found to be highly reliable among independent assessors. Using AQUA-12, we found that the majority of ADR reports are of high quality. However, important details were missing in a large proportion of reports for certain data elements, such as the ADR narrative, and the description of the actual reaction, which may impact on causality assessment and further management recommendations. AQUA-12 further underscored that the identification of suspected medications is an area that requires further attention for improvement.

A recent review highlighted that large variations exist for ADR management and reporting in hospital settings [10]. It also found that current quality assessment tools for ADR reports are not standardised across the board and are also unnecessarily complex with requirements for a vast array of data. The most widely used criteria, VigiGrade, also consists of a scoring system that is counterintuitive to causality assessment, where points can be assigned to medications started after the onset of the reaction [12]. Furthermore, all available tools have been utilised only in research settings at pharmacovigilance centres. To date, there is no quality assessment tool that is both pragmatic and easily applicable to daily clinical practice in local hospital settings.

Quality improvement methods utilise research tools where process and outcome measures can be monitored in a frequent manner to rapidly detect changes in trends, variations and process limits over time [13]. Adhering to this principle, the ease of application of AQUA-12 provides a distinct advantage, compared to other methods such as questionnaires and surveys, by being able to systematically and reliably monitor ADR reports on a regular basis to detect changes in quality, resulting from any interventions introduced to the reporting processes, such as education programs [13]. The inter-rater reliability of AQUA-12 was excellent; it was robustly tested through ADRRC members from different specialties and also by the inclusion of three independent assessors external to the ADRRC review processes who are at different levels of seniority (two junior medical doctors and a clinical pharmacologist). Furthermore, AQUA-12 was designed to assess the practical application of knowledge and skills of reporting healthcare professionals through the completeness of information provided in ADR reports. Data elements in AQUA-12 are based on ADR principles and closely reflect the information required in the hospital ADR report form, which adheres to the reporting requirements by the Therapeutic Goods Administration, the national pharmacovigilance authority in Australia [14, 15].

Previous studies at our institution found that approximately 85% of ADRs were reported to ADRRC by hospital pharmacists, a rate similar to the current study [11]. Further, significant knowledge gaps exist among healthcare professionals regarding ADR principles that are important to assessment and management, particularly that of ADR syndrome recognition and causality attribution [14]. In keeping with these findings, the current study using the AQUA-12 tool also noted similar knowledge deficiencies, where reporters omitted detailed information regarding the actual reaction and description of key events around the ADR narrative (i.e. information relevant to syndrome recognition/diagnosis) and identification of suspected medications, which is important in causality attribution. In this study, we also attempted to identify if there might be specific drivers behind low-quality reports (as defined by AQUA-12 score of < 10) and found that there was a higher proportion of mild and fatal reactions, or reactions of unrecorded severity in reports that were deemed low quality. We postulate that attitudinal factors, such as diffidence and ignorance [16], may have contributed to the lack of effort in compiling a high-quality report, especially for mild or fatal reactions where perceived importance to subsequent patient management may have been diminished.

To address these knowledge gaps and attitudes, a multidisciplinary, multi-modular, interactive education program is currently being developed as a quality improvement initiative, to commence in late 2022. This education program will deliver the content in the following modules: (i) classification of ADRs, (ii) practical skills on recognition and diagnosis of common ADRs, (iii) basic immunological mechanisms and type B (allergic reactions) ADR pathogenesis, (iv) how to conduct a comprehensive causality assessment, (v) how to report an ADR (including professional responsibilities and attitudinal factors that influence reports) and (vi) providing risk communication to patients. One of the main applications of AQUA-12 will be to assess for any improvement in quality scores after each education module has been implemented.

The main limitation of this study is that the AQUA-12 tool was developed and evaluated at a single institution, hence, its generalisability may be limited. Nevertheless, the tool was developed specifically for quality improvement purposes within our institution, and it has been shown to be highly reliable for its intended function. Further to that, as the data elements of the AQUA-12 tool are based on key ADR principles and reporting requirements by the national pharmacovigilance authorities, a tool of similar nature could easily be adapted to suit the quality improvement initiatives at other institutions. Secondly, similar to a previous study, we observed preferential reporting of immunologically mediated ADRs (~ 80%) at our institution, while it is known that non-immunologically mediated reactions constitute a larger proportion of ADRs [17]. Further prospective evaluation over time is thus warranted to monitor the quality of reporting of non-immunologically mediated reactions.

## Conclusion

This study demonstrated that AQUA-12 is a practical quality assessment tool that can be utilised in hospital settings to regularly monitor the completeness of ADR reports. Using AQUA-12, data elements decreasing the quality of ADR reports have been identified, which will guide quality improvement efforts through an ADR education program.

## Data Availability

Data sharing is not available.
